# Protein Phosphatase-1 Activates CDK9 by Dephosphorylating Ser175

**DOI:** 10.1371/journal.pone.0018985

**Published:** 2011-04-21

**Authors:** Tatiana Ammosova, Yuri Obukhov, Alexander Kotelkin, Denitra Breuer, Monique Beullens, Victor R. Gordeuk, Mathieu Bollen, Sergei Nekhai

**Affiliations:** 1 Center for Sickle Cell Disease, Howard University, Washington, D.C., United States of America; 2 RCMI Proteomics Core Facility, Howard University, Washington, D.C., United States of America; 3 Department of Microbiology, Howard University, Washington, D.C., United States of America; 4 Department of Molecular Cell Biology, Catholic University of Leuven, Leuven, Belgium; Queensland Institute of Medical Research, Australia

## Abstract

The cyclin-dependent kinase CDK9/cyclin T1 induces HIV-1 transcription by phosphorylating the carboxyterminal domain (CTD) of RNA polymerase II (RNAPII). CDK9 activity is regulated by protein phosphatase-1 (PP1) which was previously shown to dephosphorylate CDK9 Thr186. Here, we analyzed the effect of PP1 on RNAPII phosphorylation and CDK9 activity. The selective inhibition of PP1 by okadaic acid and by NIPP1 inhibited phosphorylation of RNAPII CTD *in vitro* and *in vivo*. Expression of the central domain of NIPP1 in cultured cells inhibited the enzymatic activity of CDK9 suggesting its activation by PP1. Comparison of dephosphorylation of CDK9 phosphorylated by (^32^P) *in vivo* and dephosphorylation of CDK9's Thr186 analyzed by Thr186 phospho-specific antibodies, indicated that a residue other than Thr186 might be dephosphorylated by PP1. Analysis of dephosphorylation of phosphorylated peptides derived from CDK9's T-loop suggested that PP1 dephosphorylates CDK9 Ser175. In cultured cells, CDK9 was found to be phosphorylated on Ser175 as determined by combination of Hunter 2D peptide mapping and LC-MS analysis. CDK9 S175A mutant was active and S175D – inactive, and dephosphorylation of CDK9's Ser175 upregulated HIV-1 transcription in PP1-dependent manner. Collectively, our results point to CDK9 Ser175 as novel PP1-regulatory site which dephosphorylation upregulates CDK9 activity and contribute to the activation of HIV-1 transcription.

## Introduction

CDK9/cyclin T1 is a protein kinase that phosphorylates the C-terminal domain (CTD) of the largest subunit of RNA polymerase II (RNAPII) [1]. CDK9/cyclin T1 is found within large and small molecular weight complexes. The large complex contains 7SK RNA [2], [3], the hexamethylene bisacetamide (HEXIM1) protein [4], [5] and recently identified La-related LARP7 protein [6], [7], [8] and methylphosphatase capping enzyme MePCE [9], [10]. The activity of CDK9 in the large complex is inhibited by interaction with 7SK RNA and HEXIM1 [2], [3], [4], [5]. In contrast, the small complex is devoid of 7SK RNA and HEXIM1 protein, and contains enzymatically active CDK9 [2], [3]. Association of CDK9/cyclin T1 with 7SK RNA/HEXIM1 is mediated by phosphorylation of CDK9 on Thr186, which is also required for the enzymatic activity of CDK9 [11], [12]. In addition to Thr186, several serine and threonine residues in CDK9 can be phosphorylated. These residues include N-terminal Thr29 [13]; T-loop Ser175 [12], [14], and the C-terminal residues Ser329, Thr330, Thr333, Ser334, Ser347, Thr350, Ser353, and Thr354, collectively referred to as ‘C8’ [12], [15]. The Thr29 and C8 residues are autophosphorylated [12], [13], [15]. Earlier *in vitro* studies showed that CDK9 autophosphorylation on C8 residues was prerequisite for the binding of CDK9/cyclin T1 to TAR RNA [15], [16]. Previously, we showed that CDK9 autophosphorylated *in vitro* is dephosphorylated by protein phosphatase 2A (PP2A) but not protein phosphatase-1 (PP1) [17]. Also, PP2A but not PP1 prevented the binding of CDK9/cyclin T1 to TAR RNA and Tat [17] confirming previous observation that CDK9 autophosphorylation is needed for its binding to TAR RNA [15], [16]. Contrary to the strong CDK9 dephosphorylation by PP2A *in vitro*, inhibition of PP2A *in vivo* had little effect on CDK9 phosphorylation and HIV-1 transcription which is sensitive to CDK9. On the other hand, inhibition of PP1 *in vivo* strongly increased CDK9 phosphorylation and profoundly inhibited Tat-activated HIV-1 transcription [17]. The strong effect of PP1 *in vivo* suggested that unlike the situation *in vitro*, CDK9 might be dephosphorylated by PP1 in the cells and that PP1 might have a regulatory role in HIV-1 transcription. Indeed, we previously showed that PP1 expression induced HIV-1 transcription in cultured cells [18] and expression of PP1 inhibitor, NIPP1, inhibited Tat-induced HIV-1 transcription and replication [18], [19], [20]. Based on our previous studies we hypothesized that PP1 might dephosphorylate CDK9 residue(s) other that those shown to be autophosphorylated *in vitro*, i.e Thr29 and C8 residues. One plausible candidate for PP1 dephosphorylation is CDK9 T-loop that includes Ser175 and Thr186 residues. Recently, CDK9 activation in stress-induced cells was shown to be cooperatively induced by PP1α and protein phosphatase 2B (PP2B) [21]. PP1α was shown to dephosphorylate Thr186 and disrupt the interaction between CDK9/cyclin T1 and 7SK RNA/HEXIM1 [21]. In the present paper, we show that PP1 inhibition in cell extracts *in vitro* and in cultured cells reduced RNAPII CTD phosphorylation and the activity of CDK9, which indicated that PP1 is an activator of CDK9. Comparison of dephosphorylation of CDK9 phosphorylated by (^32^P) *in vivo* and dephosphorylation of CDK9's Thr186 analyzed by Thr186 phospho-specific antibodies, indicated that a residue other than Thr186 might be dephosphorylated by PP1. Analysis of dephosphorylation of phosphorylated peptides derived from CDK9's T-loop and also *in vivo* phosphorylated CDK9 with Ser175 mutation showed that Ser175 could be dephosphorylated by PP1 *in vitro*. Further analysis of CDK9 phosphorylation residues was carried by Hunter 2D peptide mapping and LC-MS, pointing to CDK9 Ser 175 as one of the two phosphorylation sites. Analysis of the activity of CDK9's S175A and S175D mutants showed that CDK9 S175A mutant was active and S175D - inactive. Expression of cdNIPP1 prevented the interaction of CDK9 S175D with HEXIM1. Analysis of the effect of CDK9 point mutations alone or in combination with PP1 on HIV-1 transcription which is dependent on CDK9, indicate that dephosphorylation of CDK9's Ser175 upregulated HIV-1 transcription in PP1-dependent manner. Collectively, our results point to Ser175 as PP1-regulatory site in CDK9 which dephosphorylation upregulates CDK9 activity and may contribute to the regulation of HIV-1 transcription.

## Results

### Inhibition of PP1 prevents RNAPII CTD phosphorylation *in vitro*

**Figure 1:**
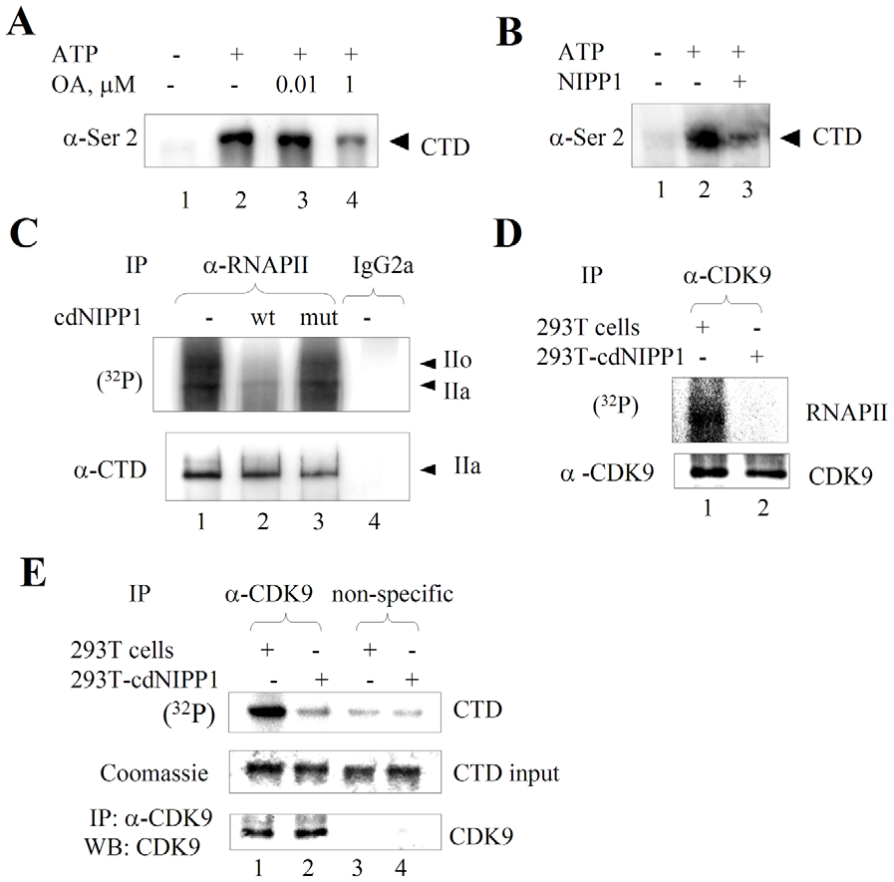
Inhibition of PP1 prevents RNAPII CTD phosphorylation and inhibits CDK9 activity. (**A**) **High concentration of okadaic acid inhibits RNAPII phosphorylation** ***in vitro*** **.** HeLa cell nuclear extract was subjected to *in vitro* transcription without (lane 2) or with the addition of 10 nM okadaic acid (lane 3) or 1 µM okadaic acid (lane 4). Lane 1, untreated HeLa cell nuclear extract. RNAPII was resolved on 5% SDS-PAGE and analyzed with RNAPII CTD serine 2 phospho-epitope specific antibodies (Ser2). (**B**) **NIPP1 prevents RNAPII phosphorylation** ***in vitro***. HeLa cell nuclear extract was subjected to *in vitro* transcription without (lane 2) or with the addition of 5 µM NIPP1 (lane 3). Lane 1, untreated HeLa cell nuclear extract. RNAPII was resolved on 5% SDS-PAGE and analyzed with Ser2 phospho-epitope specific antibodies. (**C**) **Expression of cdNIPP1 prevents RNAPII phosphorylation in cultured cells.** 293T cells were transfected with vectors expressing wt cdNIPP1 (lane 2) or mutant cdNIPP1 (lane 3) or mock transfected (lane 1). At 48 hours post transfection, the cells were treated with 0.1 µM okadaic acid and pulsed with (^32^P) orthophosphate for 3 hours. The cellular lysates were subjected to immunoprecipitation with 8WG16 antibodies against RNAPII CTD (lanes 1 to 3) or with non-specific mouse IgG2a (lane 4). Immunoprecipitated RNAPII was resolved on 5% SDS-PAGE and the gel was analyzed on Phosphor Imager. Separately, RNAPII was immunoprecipitated and analyzed by Western blotting (lower panel). (**D & E**) **Expression of cdNIPP1 inhibits enzymatic activity of CDK9.** Lysates of 293T cells (lane 1) or 293T cells continuously expressing cdNIPP1 (293T-cdNIPP1 cells) (lane 2) were immunoprecipitated with anti-CDK9 antibodies. Precipitated CDK9 was supplemented with γ-(^32^P) ATP and purified yeast RNAPII (panel D) or GST-CTD (panel E) as substrates. GST-CTD and RNAPII were resolved on 10% and 7.5% SDS-PAGE gels and the gels were analyzed on Phosphor Imager. Immunoprecipitation of CDK9 was verified by immunoblotting (lower panel D). Also there was phosphorylation in the absence of substrate ((lower panel E) or when non-specific antibodies were used (panel E, lanes 3 and 4). Results are from a typical experiment of 2–4 performed.

We previously showed that after the inhibition of RNAPII CTD kinases by flavopiridol, the RNAPII CTD is quickly dephosphorylated by PP1 [22]. Here, we conducted an opposite experiment, that is we inhibited PP1 and then analyzed RNAPII CTD phosphorylation. RNAPII was phosphorylated in an *in vitro* transcription reaction and its phosphorylation was visualized by immunoblotting with phospho-epitope specific monoclonal antibodies that recognized phospho Ser-2 residues in the heptapeptide repeats of the CTD (Fig. 1A, compare lanes 1 and 2). Addition of 10 nM okadaic acid, a concentration inhibitory for PP2A [17], did not change RNAPII CTD phosphorylation (Fig. 1A, lane 3). In contrast, a strong decrease in the phosphorylation of the CTD of RNAPII was observed in the presence of 1 µM okadaic acid, which is inhibitory to both PP2A and PP1 (Fig. 1A, lane 4), suggesting that inhibition of PP1 in the nuclear extract inhibited an RNAPII-directed kinase. To verify that the inhibition of RNAPII phosphorylation was due to the inhibition of PP1, recombinant NIPP1, a potent and specific inhibitor of PP1 [22], was added to the nuclear extract. This resulted in the reduction of Ser-2 phosphorylation of RNAPII (Fig. 1B, lane 3). These results demonstrated that specific inhibition of PP1 in nuclear extract reduced the level of RNAPII phosphorylation and this is more likely the inhibition of a kinase rather than the direct RNAPII CTD dephosphorylation of PP1.

### Inhibition of PP1 prevents RNAPII CTD phosphorylation in cultured cells

We extended the *in vitro* observation to the cultured cells by examining the effect of PP1 inhibition on the dynamic RNAPII phosphorylation. The RNAPII phosphorylation was measured by the incorporation of radioactive (^32^P) into the RNAPII in cultured cells. 293T cells were transfected with vectors expressing an inhibitory fragment of NIPP1 (cdNIPP1 wt) or the corresponding PP1 binding mutant (V201A/F203A, mut) [23]. At 24 hours post-transfection, cells were pulsed with (^32^P) orthophosphate for 3 hrs and also treated with 0.1 µM okadaic acid to inhibit PP1. RNAPII was immunoprecipitated from the cell lysates and its phosphorylation was analyzed. Expression of cdNIPP1 reduced the incorporation of (^32^P) into RNAPII (Fig. 1C, lane 2) in comparison to the mock-transfected or cdNIPP1 mut transfected cells (Fig. 1C, lanes 1 and 3). Thus, the inhibition of PP1 affects the phosphorylation of RNAPII CTD both *in vitro* and in cultured cells.

### PP1 dephosphorylates CDK9 that was metabolically labeled with (^32^P) in cultured cells

**Figure 2:**
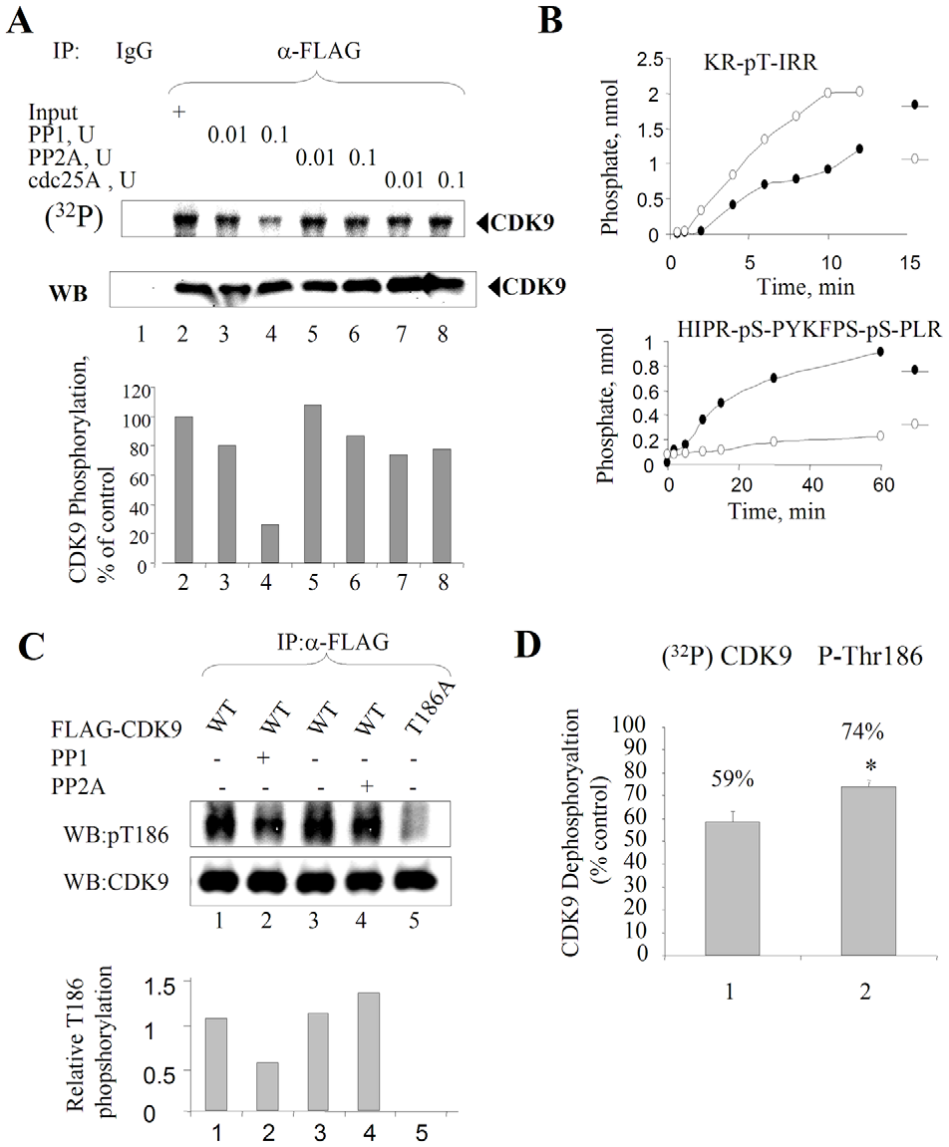
Dephosphorylation of *in vivo* labeled CDK9 by PP1. (**A**) **PP1 dephosphorylates CDK9 (^32^P)-phosphorylated in cultured cells.** 293T cells transfected with Flag-CDK9 vector were pulsed with (^32^P) orthophosphate in the presence of 100 nM okadaic acid. Flag-CDK9 was immunoprecipitated with anti-Flag antibodies (lane 2) and subjected to dephosphorylation with PP1 (lanes 3 and 4), PP2A (lanes 5 and 6) or cdc25A (lanes 7 and 8). Lane 1, immunoprecipitation with non-specific mouse IgG. The reactions were resolved on 10% SDS-PAGE and analyzed on Phosphor Imager. (**B**) **Comparison of phosphatase activities of PP1 and PP2A.** Recombinant PP1 (0.1 U) and purified from human red blood cells PP2A (0.1 U) were assayed with the generic KR-pT-IRR substrate or phospho-Rb peptide and the reactions were stopped at indicated time points by the addition of malachite green solution. The amount of malachite green was quantified by the absorbance and recalculated into the phosphate concentration using phosphate standard curve. (**C**) **Dephosphorylation of CDK9's Thr186 by PP1.** 293T cells were transfected with vectors expressing Flag-CDK9 WT (lanes 1–4) or Flag-CDK9 T186A (lane 5) and treated at 48 hours posttransfection with 100 nM okadaic acid. CDK9 was immunoprecipitated from cellular lysates with anti-Flag antibodies, subjected to dephosphorylation with PP1 (lane 2) or PP2A (lane 4) and analyzed by immunoblotting with phospho-specific CDK9 Thr186 or anti-CDK9 antibodies. (**D**) **Comparison of dephosphorylation of (^32^P) CDK9 and Thr186-phosphorylated.** Combined results from three independent experiments shown as percent of CDK9 dephosphorylation. *P<0.05. Results are from a typical experiment of 4 performed.

We previously showed that inhibition of PP1 in cultured cells increased CDK9 phosphorylation [17], suggesting that PP1 may dephosphorylate CDK9. To analyze CDK9 dephosphorylation directly, we metabolically labeled endogenous CDK9 *in vivo*. FLAG-tagged CDK9 was expressed in 293T cells and phosphorylated by a pulse with (^32^P) orthophosphate and also by the addition of 0.1 µM okadaic acid to inhibit cellular PP1 [17]. The (^32^P)-phosphorylated FLAG-CDK9 was immunoprecipitated and subjected to dephosphorylation by PP1, PP2A and, as a control, Cdc25, a dual-specificity phosphatase that dephosphorylates Thr14 and Tyr15 residues of CDK2 [24]. Phosphorylated CDK9 was efficiently dephosphorylated by PP1, but not by PP2A or Cdc25A (Fig. 2A). Because recombinant phosphatases *in vitro* may not be specific toward their substrates, we compared PP1 and PP2A with a commercially available substrate, KR-pT-IRR peptide (Millipore) or a specially designed PP1 substrate, phospho-Rb peptide (HIPR-pS-PYKFPS-pS-PLR). While PP1 and PP2A both dephosphorylated the KR-pT-IRR peptide with PP2A being somewhat more efficient (Fig. 2B), only PP1 dephosphorylated the phospho-Rb peptide (Fig. 2B), confirming that PP1 and PP2A displayed distinct substrate specificity. Thus PP1 and not PP2A specifically dephosphorylated CDK9 that was phosphorylated *in vivo*.

### PP1 targets other residues of CDK9 in addition to Thr186

To analyze whether Thr186 of CDK9 was the only target site for PP1 dephosphorylation, FLAG-CDK9 wild type (WT) or the T186A mutant were expressed in 293T cells. Subsequently, the cells were treated with okadaic acid, CDK9 was immunoprecipitated with anti-FLAG antibodies from the cell lysates and the immunoprecipitates were subjected to dephosphorylation by PP1 or PP2A. In contrast to the almost complete dephosphorylation of (^32^P)-labeled CDK9 by PP1 (Fig. 2A), dephosphorylation of Thr 186 was partial (Fig. 2C, lane 2), suggesting that PP1 might target additional phosphorylation sites in CDK9. Combined results from independent experiments show that (^32^P)-labeled CDK9 is more efficiently dephosphorylated by PP1 than the Thr186-phosphorylated CDK9 (Fig. 2D).

### PP1 dephosphorylates CDK9 on Ser 175

**Figure 3:**
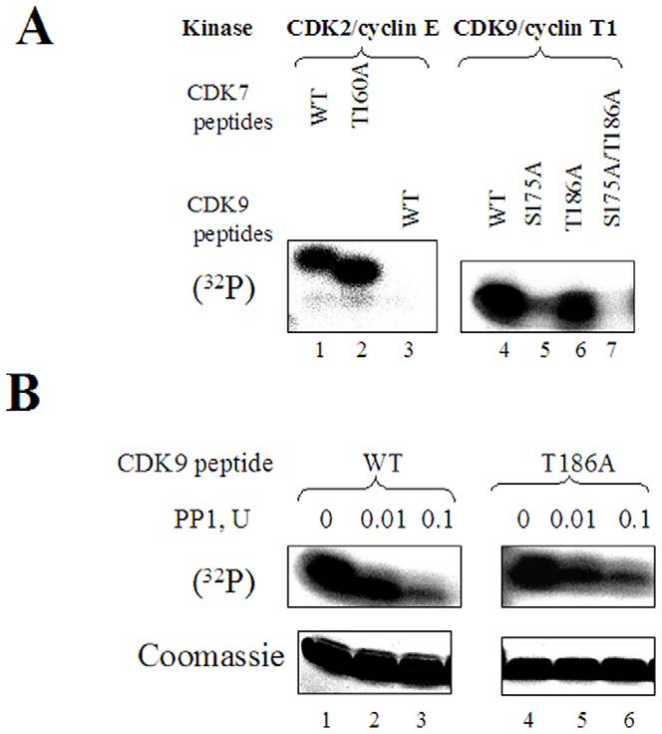
PP1 dephosphorylates T-loop derived CDK9 peptide phosphorylated on Ser 175. (**A**) **Phosphorylation of CDK9's T-loop derived peptides.** CDK7 or CDK9 T-loop derived peptides were phosphorylated by recombinant CDK2/cyclin E or CDK9/cyclin T1, resolved on 15% SDS Tric-Tricine gel and analyzed by Phosphor Imager. Lanes 1 and 2, phosphorylation of CDK7 derived T-loop peptides by CDK2/cyclin E. Lanes 4–7, phosphoryaltion of WT and mutant CDK9's T-loop-derived peptides by recombinant CDK9/cyclin T1. (**B**) **Dephosphorylation of CDK9's T-loop derived peptides by PP1.** CDK9-derived T loop WT peptides (lanes 1–3) or T186A mutant peptides (lanes 4–6) were phosphorylated by recombinant CDK9/cyclin T1, then CDK9 activity was blocked by 10 µM ARC and the peptides were incubated with the indicated amount of PP1. The peptides were resolved on 15% Tris-Tricine gel and analyzed by Phosphor Imager (upper panel) and also showed stained with Coomassie Blue (lower panel). Results are from a typical experiment of 3 performed.

We analyzed dephosphorylation of CDK9's T-loop using a synthetic peptide that spans the T-loop of CDK9 (^172^ RAFSLAKNSQPNRYTNRVV ^190^) and comprised both Ser175 and Thr 186 residues. We also used alanine mutants of Ser175 (S175A: ^172^ RAFALAKNSQPNRYTNRVV ^190^), Thr186 (T186A: ^172^ RAFSLAKNSQPNRYANRVV ^190^) or both residues (S175A/T186A: ^172^ RAFALAKNSQPNRYANRVV ^190^). The T-loop-derived WT CDK9 and CDK9 T186A peptides were efficiently phosphorylated by whole cell extracts whereas the S175A peptide and the double mutated S175A/T186A peptide were not phosphorylated suggesting that Ser175 but not Thr186 kinase activity was present abundantly in the cell extract (data not shown). Previously, CDK9/cyclin T1 was shown to autophosphorylate its T-loop [25]. Also, CDK2/cyclin E is known to phosphorylate the T-loop of CDK7 [26]. Indeed, recombinant CDK2/cyclin E efficiently phosphorylated the T-loop peptides of CDK7 (WT: ^150^ KSFGSPNRIYTHQVV ^165^; T160A: ^150^ KSFGSPNRIYAHQVV ^165^) (Fig. 3A, lanes 1 and 2), but was not able to phosphorylate the T-loop of CDK9 (Fig. 3A, lane 3). In contrast, recombinant CDK9/cyclin T1 phosphorylated both WT and the T186A mutant peptides (Fig. 3A, lanes 4 and 6), but to a lesser extent the S175A peptide (Fig. 3A, lane 5). CDK9/cyclin T1 did not phosphorylate the S175/T186A mutant (Fig. 3A, lane 7) ruling out the phosphorylation of an additional serine residue, Ser180, that is also present in the T-loop peptide. To analyze the dephosphorylation of Ser175 by PP1, WT or T186A mutant T-loop peptides were first phosphorylated by recombinant CDK9/cyclin T1, then the CDK9 activity was blocked by the addition of the CDK9 inhibitor ARC (1 µM, [27]) (Fig. 3B, lanes 1 and 4). Dephosphorylation of the CDK9-phosphorylated WT and T186A peptides showed that both of these peptides were efficiently dephosphorylated by PP1 (Fig. 3B, lanes 2, 3, 5 and 6). Together these results suggest that Ser175 is efficiently phosphorylated by an abundant kinase activity that may include CDK9/cyclin T1 and that Ser175 is dephosphorylated by PP1 *in vitro*.

### CDK9 is phosphorylated on Ser175 *in vivo*

**Figure 4:**
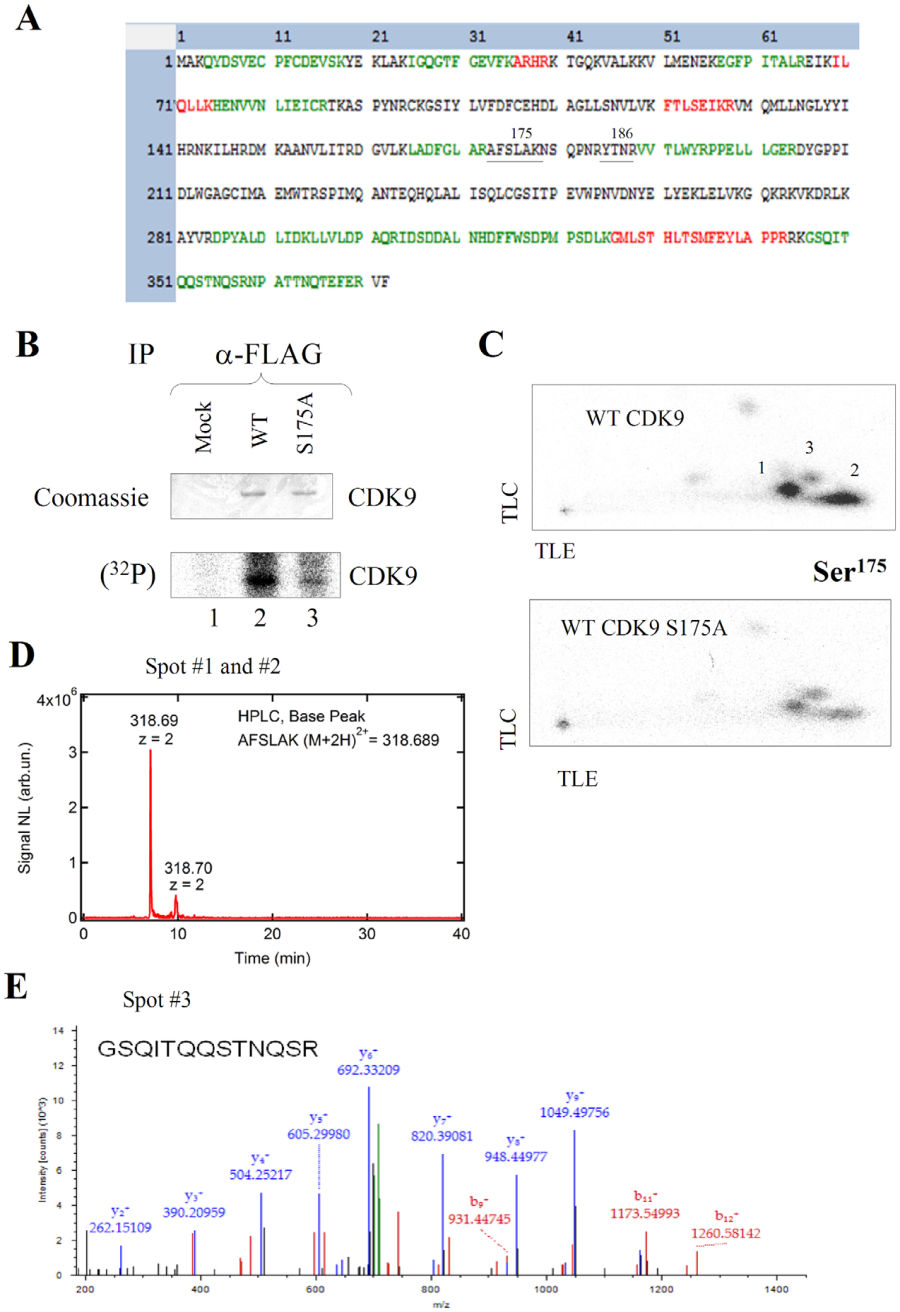
CDK9 is phosphorylated on Ser175 residue in cultured cells. (**A**) **MS/MS analysis of recombinant CDK9.** Recombinant CDK9/cyclin T1 was resolved on 10% SDS-PAGE. CDK9 was identified by Coomassie staining, in-gel digested with trypsin, and the eluted peptides were subjected to MS analysis on Thermo LTQ Orbitrap XL mass spectrometer as described in Materials and Methods. The SEQUEST search results are shown. Green, peptides identified with high probability by MS/MS analysis. Red, peptides identified with less probability. Black, peptides that were not detected. (**B**) **purification of (^32^P)-labeled CDK9 for the peptide fingerprint analysis.** FLAG-tagged CDK9 was expressing in 293T cells and metabolically labeled in the presence of okadaic acid. CDK9 was immunoprecipitated, resolved on 10% SDS-PAGE and stained with colloidal Coomassie (upper panel), or exposed to Phosphor imager screen lower panel. Lane 1, mock-transfected cells. Lane 2, WT CDK9. Lane 3, CDK9 S175A mutant. (**C**) **Tryptic phosphopeptide mapping.** (^32^P)-labeled CDK9 was trypsinized and resolved on Hunter thin layer peptide mapping electrophoresis system as described in Materials and Methods. WT CDK9 (upper panel) and CDK9 S175A (lower panel) are shown. Spots labeled as 1–3 were scraped and further analyzed by MS analysis. The results are representative from 2 experiments. (**D**) **Base peak chromatography of Spot 1.** Raw base peak chromatography data showing ion with mass 318.69 that matches to AFSLAK (M+2H)^2+^ peptide. Results are representative from 4 experiments. **E**. **MS/MS spectrum of derived from Spot 3.** The spectrum gives positive identification of GSQITQQSTNQSR peptide. Results are from a typical experiment of 3 performed.

Previously, analysis of recombinant CDK9 revealed phosphorylation of Thr186 [25], [28]. Interestingly, in both reports, only partially hydrolyzed NSQPNRYpT^186^NR peptide was detected whereas fully hydrolyzed YpT^186^NR peptide was not seen. We conducted LC-MS/MS analysis using Thermo LTQ Orbitrap XL connected to the nano-LC to determine the possibility of detection of T-loop derived peptides. Analysis of recombinant CDK9/cyclin T1 by MS/MS analysis showed good coverage of the CDK9 sequence, but the T-loop peptides were not detected (Fig. 4A). Because ionization efficiency strongly depends on the peptide structure, some peptides in principle may be “invisible” for the MS analysis. Thus, we resorted to the Hunter peptide thin layer electrophoresis followed by chromatography that resolves well (^32^P) phosphorylated peptides [29]. FLAG-tagged CDK9 was expressed in 293T cells, metabolically labeled with (^32^P) in the presence of 0.1 µM okadaic acid and then immunoprecipitated with anti-FLAG antibodies (Fig. 4B). CDK9 detected by Coomassie staining and also by radioactivity was excised and in-gel trypsinized. Tryptic peptides resolved by Hunter peptide mapping system showed two major spots and one minor spot (Fig. 4C, spots 1–3). The intensity of the spots 1 and 2 was greatly reduced in the CDK9 S175A mutant (Fig. 4C) suggesting that at least one spot contains Ser 175 phosphorylated peptide. We further analyzed spots 1–3 by scraping the cellulose extracting the peptides and subjecting them to the MS analysis. We detected ions with the mass coinciding for the AFS^175^LAK peptide as shown by the elution of 318.69 Da ion in the raw base peak chromatography data (Fig. 4D). The MS/MS data of this peak did not have enough intensity to be positively identified by SEQUEST search. The spot #3 showed MS/MS spectrum that allowed positive identification of ^346^ GSQITQQSTNQSR ^358^ peptide (Fig. 4E). To determine if Spot#2 is a partially hydrolyzed Ser175-containing peptide, we scraped the spot, trypsinized it overnight and rerun on the Hunter system. After re-trypsinization, the Spot 2 migrated on the plate as Spot 1 suggesting that indeed Spots 1 and 2 were the same phosphopeptides. Taken together, these data strongly indicated that CDK9 is phosphorylated on Ser175 in cultured cells.

### Mutation of Ser175 prevents CDK9 dephosphorylation by PP1

**Figure 5:**
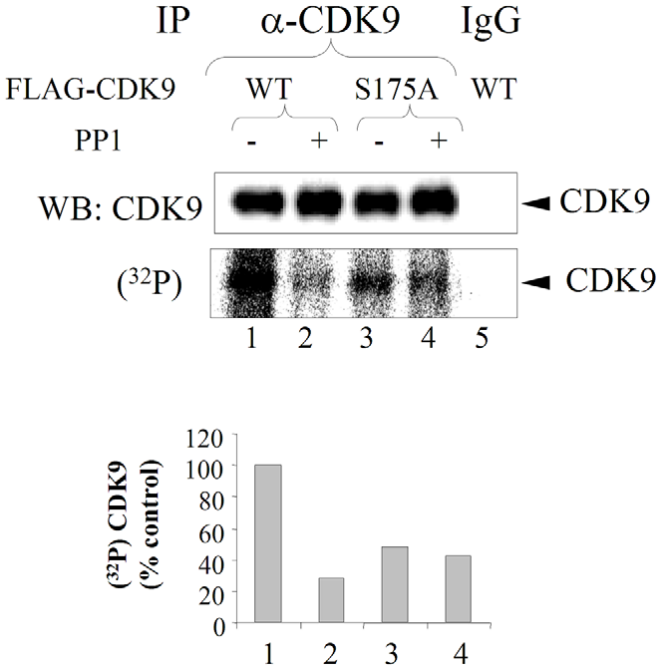
PP1 does not dephosphorylate CDK9 S175A mutant. 293T cells were transfected with vectors expressing Flag-tagged CDK9 WT (lanes 1, 2 and 5) or CDK9 S175A mutant (lanes 3 and 4) and treated at 48 hours posttransfection with 100 nM okadaic acid and (^32^P) orthophosphate. CDK9 was immunoprecipitated from cellular lysates with anti-Flag antibodies and subjected to dephosphorylation by PP1 as indicated. Lane 5, immunoprecipitation with non-specific mouse IgG. The reactions were resolved on 10% SDS-PAGE and analyzed on Phosphor Imager and by immunoblotting with anti-CDK9 antibodies. On a lower panel quantitation of (^32^P) phosphorylation of CDK9 is shown. Results are from a typical experiment of 2 performed.

We further analyzed the dephosphorylation of CDK9's Ser175 by utilizing WT CDK9 and CDK9 S175A mutant that were expressed as FLAG-tagged constructs in cultured cells, labeled with (^32^P) orthophosphate in the presence of okadaic acid and subjected to immunoprecipitation and dephosphorylation by PP1. Phosphorylated CDK9 was efficiently dephosphorylated by PP1 (Fig. 5, lanes 1 and 2). Mutation of CDK9 in Ser175 reduced overall level of CDK9 phosphorylation and but also made CDK9 S175A mutant insensitive to PP1 (Fig. 5, lanes 3 and 4). This result suggests that Ser175 is targeted by PP1.

### CDK9 S175A is enzymatically active and CDK9 S175D is inactive

**Figure 6:**
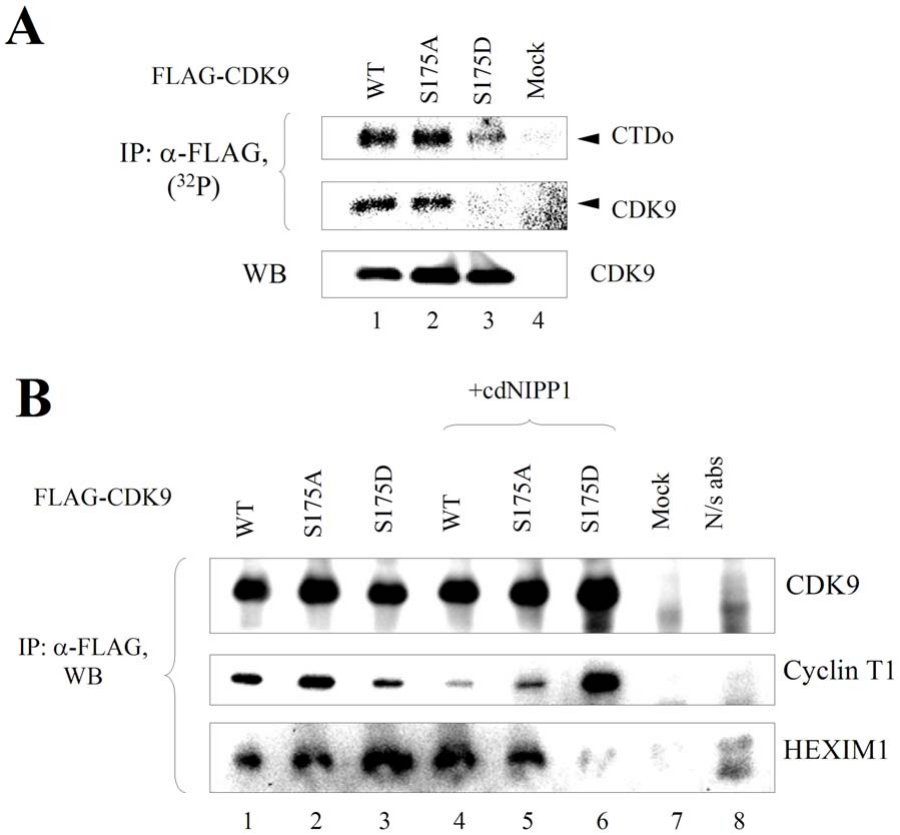
CDK9 S175D mutation inhibits CDK9 kinase activity. 293T cells were transfected with the vectors expressing FLAG-tagged CDK9 WT, CDK9 S175A or S175D mutants along with the cyclin T1-expression vector (panel A) or with the inclusion of cdNIPP1 expression vector (panel D). At 48 hours posttransfection, CDK9 was immunoprecipitated with anti-FLAG antibodies and analyzed for its kinase activity with GST-CTD substrate (panel A) or by immunoblotting (panel B) with anti-CDK9, anti-cyclin T1 antibodies and HEXIM1 antibodies. Results are from a typical experiment of 3 performed.

We next analyzed the effect of CDK9 Ser175 phosphorylation on the activity of CDK9, using CDK9 S175A and CDK9 S175D mutants. 293T cells were transfected with vectors expressing FLAG-CDK9 (WT, S175A or S175D) and also co-transfected with cyclin T1-expressing vector. CDK9 was immunoprecipitated with monoclonal anti-FLAG antibodies and its activity was analyzed using recombinant GST-CTD as substrate. Both WT CDK9 and CDK9 S175A were active in the phosphorylation of GST-CTD and were also autophosphorylated (Fig. 6A, lanes 1 and 2). In contrast, CDK9 S175D was inactive in CTD phosphorylation and not autophosphorylated (Fig. 6A, lane 3). To determine whether the inefficient kinase activity was due to changes in the association with cyclin T1 or HEXIM1 protein, we analyzed immunoprecipitated CDK9 mutants for the presence of cyclin T1 and HEXIM1. The WT CDK9 and CDK9 mutants associated approximately to the same extent with cyclin T1 (Fig. 6B, lanes 1–3), whereas association with HEXIM1 was increased for CDK9 S175D mutant (Fig. 6B, lane 3), suggesting that the inactivity of this mutant might be due to its increased association with inhibitory 7SK snRNP. We next analyzed the association of CDK9 mutant with cyclin T1 and HEXIM1 under the condition of PP1 inhibition, which was achieved by the expression of cdNIPP1. The WT CDK9 showed decreased association with cyclin T1 and increased – with HEXIM1 (Fig. 6B, lane 4), in accord with our recent study that showed increased 7SK snRNP formation in the cells expressing cdNIPP1 [20]. While CDK9 S175A showed decreased association with cyclin T1, CDK9 S175D mutant had an increase in the cyclin T1, but surprisingly, almost no HEXIM1, in contrast to WT CDK9 and S175A mutant CDK9 (Fig. 6B, lane 6). These results indicate that Ser175 phosphorylation regulates CDK9 activity, and also association with 7SK snRNP.

### CDK9 S175A mutant activates HIV-1 transcription

**Figure 7:**
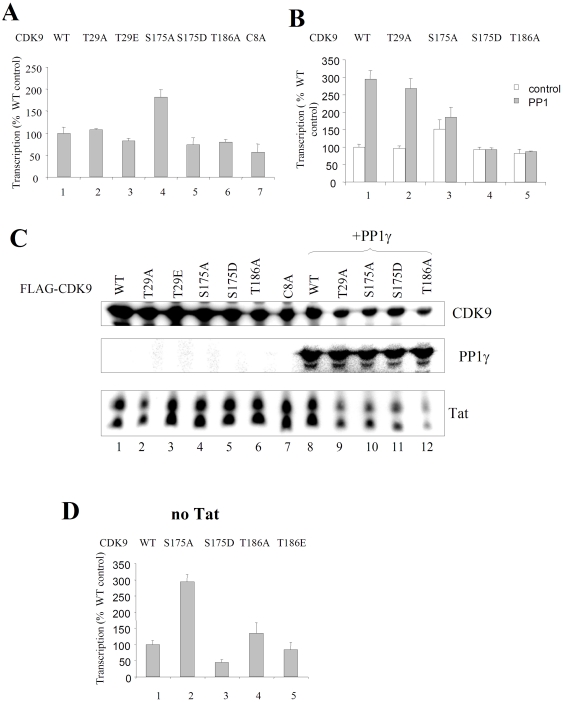
CDK9 S175A activates HIV-1 transcription. (**A**) **Analysis of CDK9 mutants for the activation of HIV-1 transcription.** 293T cells were transfected with HIV-1 LTR-LacZ and Tat expression vectors along with the indicated WT or mutated Flag- CDK9. At 48 hours posttransfection β-galactosidase activity was analyzed using ONPG substrate. Results are averages of quadruplicates from a typical experiment of 3 performed. (**B**) **PP1 induction of HIV-1 transcription is reduced in the presence of CDK9 S175A mutant.** 293T cells were EGFP (control) or PP1-EGFP expression vector in combination with indicated WT or mutated Flag-tagged CDK9 and also with HIV-1 LTR-LacZ and Tat expression vectors. At 48 hours posttransfection β-galactosidase activity was analyzed using ONPG substrate. Results are from a typical experiment of 3 performed. (**C**) **Expression of CDK9, PP1 and Tat**. Protein expression from panels A and B was verified by immunoblotting.

We previously showed that PP1 activates HIV-1 transcription [19]. If PP1 acts through the dephosphorylation of CDK9, then a non-phosphorylatable mutant of CDK9 should activate HIV-1 transcription and its effect should no longer be dependent on the presence of PP1. First, we analyzed the effect of different CDK9 mutants on Tat-induced HIV-1 transcription. Co-transfection of the CDK9 mutants with HIV-1 LTR-*LacZ* and Tat –expression vectors showed induction of HIV-1 transcription by CDK9 S175A but not any other mutants tested (Fig. 7A). We have not seen strong induction by CDK9 S175A and a strong inhibition effect by the inactive CDK9 mutants T29E, S175D, T186A and C8A (Fig. 7A), likely because the endogenous CDK9 compensated for the inactive CDK9 mutants. To determine if the induction of HIV-1 transcription by PP1 was lost with the CDK9 S175A mutant, the 293T cells were co-transfected with PP1 expression vector in combination with WT or mutant CDK9 and also HIV-1 LTR-*LacZ* and Tat –expression vectors. As a control, EGFP expression vector was used. PP1 induced HIV-1 transcription in the presence of WT CDK9 or CDK9 T29A mutant (Fig. 7B, lanes 1 and 2). In contrast, PP1 did not induced transcription beyond the induction already achieved with CDK9 S175A mutant (Fig. 7B, lane 4). Also, PP1 had no effect on HIV-1 transcription in the presence of CDK9 S175D and CDK9 T186A (Fig. 7B, lanes 5 and 6). The CDK9 T186A mutant is inactive [28] and thus may act as dominant negative to inhibit HIV-1 transcription. The CDK9 S175D is also inactive as kinase as we showed above and thus may act in a manner similar to the T186A mutant. CDK9 mutants and Tat were expressed in the absence of PP1 (Fig. 7C, lanes 1–7) and in the presence of PP1 (lanes 8–12). There was a slight reduction of Tat expression in the cells expressing CDK9 mutants and PP1 (Fig. 7C, lanes 9–12). We next analyzed the effect of CDK9 mutants on basal HIV-1 transcription. Co-transfection of the CDK9 mutants with HIV-1 LTR-*Luc* showed induction of HIV-1 transcription by CDK9 S175A (Fig. 7D).

Taken together, our results show that PP1 activates CDK9 and that this activation could be through the dephosphorylation of Ser175, mutation to alanine of which activated CDK9 enzymatic activity and induced HIV-1 transcription.

## Discussion

**Figure 8:**
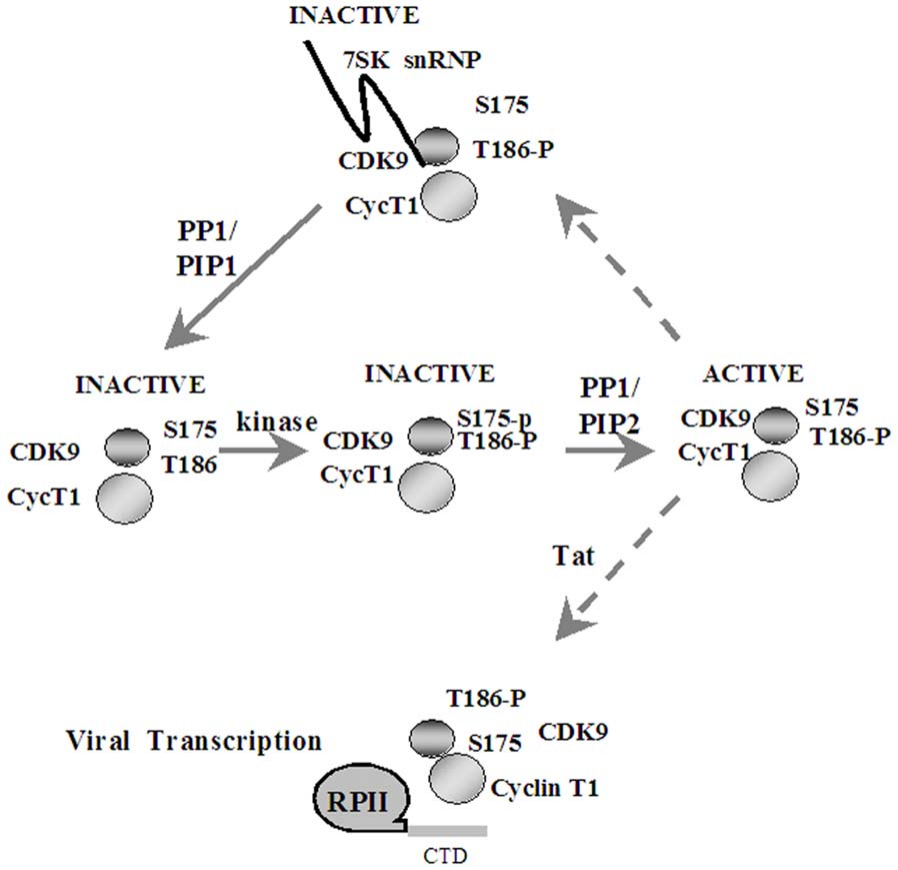
Proposed model of CDK9/cyclin T1 activation in viral transcription. CDK9's Thr186 dephosphorylation by PP1/PIP1 complex leads to the dissociation of 7SK RNA and HEXIM1 protein and the release of inactive CDK9. CDK9 is phosphorylated by a cellular kinase, which may include active CDK9/cyclin T1 on Ser175 and Thr 186 that creates inactive CDK9/cyclin T1. This inactive phosphorylated CDK9/cyclin T1 is activated by PP1/PIP2 complex. The active CDK9/cyclin T1 can be recruited by Tat or re-associated with 7SK snRNP.

In a recent study, dephosphorylation of Thr 186 by PP1α was shown to disrupt the interaction between CDK9/cyclin T1 and 7SK RNA/HEXIM1 when PP2B acted in cooperation with PP1α [21]. If Thr186 was the only residue affected by PP1, then inhibition of PP1 would increase Thr186 phosphorylation and maintain the kinase activity of CDK9. In contrast, we observed that inhibition of PP1 in nuclear extracts *in vitro* and in cultured cells inhibited RNAPII phosphorylation and downregulated CDK9 kinase activity. Our observation could not be explained on the basis of only Thr186 dephosphorylation by PP1. Comparison of the dephosphorylation of CDK9 metabolically labeled with (^32^P) in cultured cells with non-labeled CDK9 showed that residues in addition to Thr186 are likely to be dephosphorylated by PP1. Analysis of dephosphorylation of phosphopeptides and also CDK9 S175A mutant suggested that CDK9's Ser175 could be the additional dephosphorylation site. We analyzed CDK9 phosphorylation sites by combining Hunter peptide thin layer electrophoresis and LC-MS chromatography. In the cells treated with okadaic acid CDK9 was phosphorylated on two sites, which both were identified as Ser 175 containing phosphopeptides. Both of these sites were minimally phosphorylated in CDK9 S175A mutant. The third additional peptide which phosphorylation was not changed in the CDK9 S175 mutant was identified as C-terminal ^346^ GSQITQQSTNQSR ^358^ peptide. Recent study by Johnson and colleagues showed that Ser^353^ or Thr^354^ are autophosphorylated *in vitro*, in addition to the Thr^186^ residue which was also autophosphorylated [25]. Our previous study showed that CDK9 autophosphorylated *in vitro* was dephosphorylated by PP2A, but not PP1 [17]. We observed increased autophosphorylation when CDK9 was pretreated *in vitr*o with PP2A but not PP1 [17]. PP2A, but not PP1, prevented the interaction of CDK9/cyclin T1 with TAR RNA and Tat [17], which was shown previously to be dependent on the autophosphorylation of the C-terminal residues of CDK9 [15]. Thus, our present study and the previous studies indicate that PP1 is not likely to target the autophosphorylated sites of CDK9, and, according to our data, Thr186 is not likely to be autophosphorylated. While we observed phosphorylation of Ser175 peptide by CDK9, phosphorylation of the Thr186 was very poor and may be due to a contaminating kinase. Interestingly, Johnson and colleagues as well as Price and colleagues only detected partially cleaved NSQPNRYpT^186^NR peptide, but not the fully trypsinized YpT^186^NR peptide [25], [28]. Our MS/MS analysis of recombinant CDK9 showed very good coverage, but the T-loop peptides were absent. We were able to detect AFS^175^LAK peptide in the two spots resolved on Hunter thin layer electrophoresis, but were not able to perform the MS/MS analysis of the peptide. It is likely that the T-loop peptides are not well ionized and thus may not be easily detected. Previously, Morgan and colleagues detected CDK2-derived peptides by Hunter 2D peptide mapping system but noted difficulties in detection of T-loop derived peptides due to the strong binding of the peptides to the PVDF membrane [29]. A comprehensive future analysis of CDK9 phosphorylation site under different cellular conditions will need to employ more sophisticated approaches such as additional peptide modifications by MS tags or modification of the phosphate groups to create better ionized peptides. In our study, CDK9 S175A mutant was enzymatically active while CDK9 S175D mutant – inactive. Accordingly, CDK9 S175A but not CDK9 S175D mutant activated HIV-1 transcription. Our results are at variance with the previously shown negative effect of CDK9 S175A mutation on the GST-CTD phosphorylation [21] and also inability of CDK9 S175A mutant to activate HIV-1 transcription *in vitro* [30]. To note, in yet another study, CDK9 S175A and CDK9 S175D mutants were shown to be equally efficient in phosphorylating Spt5 [28]. Reasons for the contradiction between our study and other studies are not known. In stress-induced cells, CDK9/cyclin T1 dissociates from 7SK RNA and HEXIM1, and interacts with the bromodomain protein 4 (Brd4) [30], [31]. Brd4, that interacts with acetylated histones [32], recruits CDK9/cyclin T1 to various cellular promoters. Both CDK9 S175A and CDK9 S175D mutants do not bind to Brd4 [30], suggesting that phosphorylation of Ser 175 might be important for the binding of CDK9/cyclin T1 to Brd4. We recently showed that expression of cdNIPP1 disrupts the interaction of HIV-1 Tat with PP1, increases association of CDK9 with 7SK snRNP and inhibits HIV-1 transcription and replication data [20]. Our current results and recently published data are summarized in the following model shown in Figure 8. We adopted the concept of PP1 targeting by PP1-associated proteins (PIPs) [33] to propose that different PP1/PIP complexes might target PP1 to Thr 186 and Ser 175 residues of CDK9. Dephosphorylation of CDK9 Thr 186 by PP1/PIP1 helps to dissociate CDK9/cyclin T1 from 7SK snRNP [20]. The dephosphorylation creates inactive CDK9 that is unphosphorylated on Ser175 and Thr186. Phosphorylation by a cellular kinase(s) produces CDK9 phosphorylated on both Ser175 and Thr 186, which creates still inactive CDK9 because Ser175 phosphorylation inactivates CDK9, according to our study. Our analysis of phosphorylation of CDK9 peptides showed that Ser175 was much more efficiently phosphorylated in cellular extracts suggesting a possibility of preferential Ser175 over Thr186 phosphorylation. CDK9 phosphorylated on Ser 175 can be targeted by a second PP1/PIP2 complex which may or may not include Tat as Tat contains a PP1-binding motif and thus might function a PIP [34]. The dephosphorylation of CDK9 Ser175 activates CDK9/cyclin T1. The active CDK9/cyclin T1 may be recruited by Tat to activate HIV-1 transcription. Alternatively, CDK9/cyclin T1 may reassociate back with 7SK snRNP. The association with 7SK RNA was shown to require the kinase acitive CDK9 [5], [28], suggesting that Ser 175 phosphorylated CDK9. Our model explains well the effect of CDK9 S175A mutation, which increases the association of the mutant CDK9 with HEXIM1 suggesting more efficient 7SK snRNP formation. The synergetic effect of PP1 and WT CDK9 on the activation of HIV-1 transcription was not seen with CDK9 S175A mutant, suggesting that the effect of PP1 in HIV-1 transcription includes dephosphorylation of Ser 175A. Our model proposes that in the absence of PP1, CDK9 Ser175-P will not be able to associate with 7SK snRNP, the effect we observed with CDK9 S175D mutant (Fig. 7B, lane 6). We cannot, however, explain why CDK9 S175D mutant in normal conditions is seen associated with 7SK snRNP (Fig. 7B, lane 3). Perhaps, S to D mutation only partially recapitulates the effect of phosphorylation. PP1 inhibition may also cause overphosphorylation of Thr 186 or phosphorylation of additional sites in CDK9. For example, CDK9 T186E mutant does not activate HIV-1 transcription (Fig. 7) and was reported to be inactive as kinase [12], [28], suggesting S to E mutation does not mimic the effect of Thr 186 phosphorylation. Another transcriptional activator, Tax binds cyclin T1 and promotes autophosphorylation of CDK9 on Thr 29 rendering CDK9 inactive [13]. This Tax-mediated inactivation might also prevent association of CDK9/cyclin T1 with 7SK RNA and HEXIM1. In stress-induced cells, CDK9/cyclin T1 that is dissociated from the 7SK RNA/HEXIM1 protein is bound to Brd4 [30], [31]. Recently Brd4 was shown to compete with Tax for CDK9/cyclin T1 and it was proposed that Brd4 might inactivate CDK9/cyclin T1 in a manner similar to Tax thus preventing re-association of CDK9/cyclin T1 into the high molecular weight complex [35]. Previous studies showed that the Tat-bound CDK9/cyclin T1 is enzymatically active suggesting that CDK9 that is recruited by Tat is active in contrast to the Tax or Brd4 recruited CDK9/cyclin T1 [36], [37]. Further detailed studies needed to determine phosphorylation of CDK9 in the large P-TEFb complex or in complex with transcriptional activaton.

Taken together, our study uncovered CDK9's Ser 175 as new target for PP1 thus providing further details on the regulation of CDK9 activity by PP1 and suggesting PP1 for further evaluation as a new therapeutic target.

## Materials and Methods

### Materials

293T and HeLa cells were purchased from ATCC (Manassas, VA). 293T-cdNIPP1 cells stably expressing NIPP1 (143–224) were previously described [20]. PP1 was purified from rabbit skeletal muscle as previously described [38]. Recombinant PP1α was purchased from New England Biolabs (Ipswich, MA). Human protein phosphatase PP2A and cdc25A were purchased from Upstate Biotechnology (Lake Placid, NY). Human recombinant CDK9/cyclin T1 from baculovirus transfected Sf9 cells was purified as described [39]. GST-CTD was purified as described [40]. Okadaic acid and microcystin were from Sigma (Atlanta, GA). The Rb-derived phosphopeptide HIPR-pS-PYKFPS-pS-PLR (phospho-Rb) was synthesized by Synbiosci (Livermore, CA). Peptides that span T-loop of CDK9 (WT: ^172^ RAFSLAKNSQPNRYTNRVV ^190^, S175A: ^172^ RAFALAKNSQPNRYTNRVV ^190^, T186A: ^172^ RAFSLAKNSQPNRYANRVV ^190^, and S175A/T186A: ^172^ RAFALAKNSQPNRYANRVV ^190^) and peptides that span the T-loop of CDK7 (WT: ^150^ KSFGSPNRIYTHQVV ^165^; T160A: ^150^ KSFGSPNRIYAHQVV ^165^) were purchased from New England Peptides (Gardner, MA). Ser/Thr phosphatase assay kit that included KR-pT-IRR peptide was purchased from Upstate (Lake Placid, NY). Expression vectors for WT Flag-CDK9, CDK9 T175A and CDK9 T186A mutants and CDK9 with alanine substitutions of eight C-terminal serine and threonine (C8) were a gift from Dr. Qiang Zhou (University of California, Berkeley). Flag-CDK9 T29A, CDK9 T29E, CDK9 S175D and CDK9 T186E were created by mutagenesis using Quick-Change site-directed mutagenesis protocol of Stratagene (La Jolla, CA), using the appropriate primers and templates. The sequences of the DNA constructs were verified by sequencing using a commercial service from Macrogen (Rockville, MD). The expression vectors for EGFP-fused central domain of NIPP1, NIPP1-(143–224) or NIPP1-(143–224) V201AF203A _(_RATA mutant) were described [23]. NIPP1 mutated within its RVXF motif have low binding affinity for PP1 [23].

### Antibodies

Anti-CDK9 phospho-Thr186 antibodies and anti-HEXIM1 antibodies were a gift from Dr. Qiang Zhou (University of California, Berkeley). Rabbit polyclonal antibodies to CDK9, rabbit polyclonal antibodies to cyclin T1 and rabbit polyclonal antibodies to EGFP were purchased from Santa Cruz Biotechnology (Santa Cruz, CA). Anti-Flag monoclonal antibodies were purchased from Sigma (Saint Louis, MO). Monoclonal antibodies specific to the unphosphorylated form of CTD (8WG16) or CTD phosphorylated on Serine 2 (H5) were purchased from BabCo (Richmond, CA).

### CDK9 phosphorylation *in vivo* and dephosphorylation assays

293T cells were transfected with FLAG-CDK9-expression vector using Lipofectamine and Plus reagents (Life Technologies). After 48 hours incubation, the cells were placed in a phosphate-free DMEM media (Life Technologies) containing no serum for 2 hours. The media was changed to phosphate-free DMEM media supplemented with 0.5 mCi/ml of (^32^P)-orthophosphate and cells were further incubated for 3 hours at 37°C. To increase CDK9 phosphorylation, 0.1 µM okadaic acid (Sigma) was added to block cellular PPP-phosphatases. Cells were washed with PBS and lysed in a whole cell lysis buffer containing 50 mM Tris-HCl, pH 7.5, 0.5 M NaCl, 1% NP-40, 0.1% SDS and protease inhibitors cocktail (Sigma). After 10 min on ice, cellular material was scraped, incubated at 4°C for 30 min on a shaker and then centrifuged at 14,000 rpm, 4°C for 30 min. The supernatant was recovered and protein concentration was determined using Lowry protein assay (Bio-Rad). CDK9 was precipitated with monoclonal anti-Flag antibodies (Sigma) coupled to protein G-agarose for 2 h at 4°C in a TNN Buffer containing 50 mM Tris-HCl, pH 7.5, 0.15 M NaCl, and 1% NP-40. The immunoprecipitated CDK9 was washed twice with TNN buffer and then with phosphatase reaction buffer (10 mM Tris-HCl pH 7.5, 0.02 mM EDTA, 0.5 mM DTT, 5 mM caffeine, 0.01% NP-40 and 0.01% BSA) and incubated in the phosphatase reaction buffer with 0.01 or 0.1 Units of PP1, PP2A, cdc25A or non-treated (control sample) for 30 min at 30°C. Then the reactions were stopped with SDS-loading buffer, resolved on 10% SDS-PAGE (Novex, Invitrogen) and subjected to autoradiography and quantification with PhosphorImager Storm 860 (Molecular Dynamics). Analysis of CDK9 phosphorylation and dephosphorylation on Thr186 was carried out in the same way except that CDK9 phosphorylation was detected by immunoblotting with phospho-specific Thr186 antibodies.

### PP1 and PP2A dephosphorylation assays

The assays were carried out with the Ser/Thr phosphatase assay kit (Upstate). PP1 or PP2A was added in PP1 reaction buffer (50 mM HEPES pH 7.5, 100 mM NaCl, 2 mM dithiothreitol, 0.1 mM EGTA, 0.025% Tween-20) supplemented with 1 mM MnCl_2_ (New England Biolabs) that also contained 150 µM KR-pT-IRR or phospho-Rb peptide. At indicated time points, 25 µl aliquots were removed and mixed with 100 µl of Malachite Green solution. Absorbance of malachite green was determined at 620 nm and the phosphate concentration was recalculated using a calibration curve of phosphate standards prepared using 1 mM KH_2_PO_4_ solution.

### Phosphorylation of RNAPII CTD in HeLa nuclear extract

RNAPII was phosphorylated in transcription reactions (20 µl) containing 50 µg of HeLa nuclear extract, 0.5 mM ATP, CTP, UTP and GTP, 0.2 µg of JK2 [41] linearized template in buffer C (20 mM HEPES, pH 7.9, 50 mM KCl, 6.25 mM MgCl_2_, 0.5 mM EDTA, 2 mM DTT and 10% glycerol). Transcription was carried out for 30 minutes at 30°C. The reaction was terminated by addition of 7 mM EDTA.

### RNAPII labeling with (^32^P) orthophosphate and Western blots

293T cells were transiently transfected with vectors expressing EGFP-fused NIPP1 (143–224) or NIPP1 (143–224) V201AF203A (RATA mutant). At 24 hours posttransfection, the cells were pulse-labeled with (^32^P)-orthophosphate as described above and RNAPII was immunoprecipitated with anti-CTD 8WG16 monoclonal antibodies coupled to protein G agarose. The immunoprecipitated RNAPII was recovered by heating for 2 min at 100°C in SDS-loading buffer, resolved on 6% SDS-PAGE and the dried gel was exposed to Phosphor Imager screen (Packard Instruments, Wellesley, MA). In a control, the gel was transferred to a polyvinylidene fluoride (PVDF) membrane and analyzed with anti-RNAPII CTD 8WG16 antibodies.

### Phosphorylation of GST-CTD by CDK9 mutants *in vitro*

293T cells were seeded in 6-well plate and transfected with a combination of plasmids expressing CyclinT1 and FLAG-CDK9 (WT, S175A or S175D) using Lipofectamine-LTX (Invitrogen). At 48 hours posttransfection the cells were washed with PBS and lysed in a whole cell lysis buffer for 30 minutes. To precipitate chromatin the samples were centrifuged at 14,000 rpm at 4°C for 90 minutes. The lysates were equalized for protein concentration and diluted to 150 mM NaCl. CDK9 was immunoprecipitated with monoclonal anti-Flag antibodies bound to protein G agarose beads for 2 hrs at 4°C in TNN Buffer (50 mM Tris-HCl, pH 7.5, 0.15 M NaCl and 1% NP-40). The beads with precipitated CDK9 were washed with PBS and used for the kinase activity assay using GST-CTD as substrate in 50 mM HEPES-KOH buffer (pH 7.5), 10 mM MgCl_2_, 6 mM EGTA, 2.5 mM DTT, 100 µM cold ATP and 5 µCi γ-(^32^P)ATP for 30 minutes at 30°C. The reactions were stopped with Laemmli sample buffer, resolved on 10% Tris-Glycine SDS-PAGE (Novex, Invitrogen, Carlsbad, CA) and subjected to autoradiography and quantification with Phosphor Imager (Packard Instruments, Wellesley, MA).

### Phosphorylation of CDK9- and CDK7- derived peptides *in vitro*

About 2 µg of peptides that span T-loop of CDK9 or CDK7 as indicated were phosphorylated in 50 mM Hepes-KOH buffer (pH 7.5), 10 mM MgCl_2_, 6 mM EGTA, 2.5 mM DTT, 100 µM cold ATP and 5 µCi γ-(^32^P)ATP for 30 minutes at 30°C. The reactions were stopped with Laemmli sample buffer, resolved on 15% Tris-Tricine SDS-PAGE and subjected to autoradiography and quantification with Phosphor Imager (Packard Instruments, Wellesley, MA).

### Tryptic phosphopeptide mapping

Tryptic phosphopeptide mapping was conducted using Hunter thin layer peptide mapping electrophoresis system (C.B.S. Scientific, Del Mar, CA). FLAG-CDK9 was expressed in 293T cells and metabolically labeled with (^32^P) orthophosphate as described above. (^32^P)-labeled FLAG-CDK9 WT and S175A mutant were resolved on 10% SDS-PAGE. Radioactive bands were visualized using Phosphor imager and excised from the gel. The gel pieces were reduced in 10 mM DTT, alkylated with 30 mM iodoacetamide and then digested overnight with 10 ng/µl Trypsin Gold (Promega) in 50 mM ammonium bicarbonate. The eluted peptides were applied to thin layer cellulose plates (Boehringer Mannheim, Indianapolis, IN) and separated in first dimension by electrophoresis at pH 1.9 (H_2_O-acetic acid–formic acid, 900:78:22) conducted at 1000 V, 12 mA for 1 hrs and followed by ascending chromatography in Phospho-Chromatography buffer (n-butanol-pyridine-acetic acid-H2O, 75:50:15:60) for 15 hrs. Cellulose plates were dried and exposed to Phosphor imager screen. To determine the content of the tryptic phosphopeptide, cellulose containing the phosphopeptides was scraped and the peptides were eluted in 70 acetonytril with 0.1% TFA. The eluted peptides were lyophilized, resuspended in 0.1% formic acid and subjected to mass spectrometry analysis.

### Mass spectrometry

Samples were loaded to PicoFrit C18 column and eluted for 60 min with 2% to 30% gradient of acetonytrile and flow rate 300 nL/min using Shimadzu Prominence Nano HPLC. The 1 FT MS scan and 3 data dependent FT MS/MS scans were performed on Thermo LTQ Orbitrap XL mass spectrometer on major multi charged MS peaks with resolution 60000 in each event set. The resulting set of MS/MS spectra were analyzed by SEQUEST (Thermo) search with precursor and fragments tolerance 20 ppm. These high resolution data and search criteria reduce amount of false positives and dramatically decrease the search time.
